# The pseudogene DUXAP10 promotes an aggressive phenotype through binding with LSD1 and repressing LATS2 and RRAD in non small cell lung cancer

**DOI:** 10.18632/oncotarget.14125

**Published:** 2016-12-24

**Authors:** Chen-Chen Wei, Feng-Qi Nie, Li-Li Jiang, Qin-Nan Chen, Zhen-Yao Chen, Xin Chen, Xuan Pan, Zhi-Li Liu, Bin-Bin Lu, Zhao-Xia Wang

**Affiliations:** ^1^ Department of Oncology, Second Affiliated Hospital, Nanjing Medical University, Nanjing, People's Republic of China; ^2^ Department of Oncology, Haimen People's Hospital, Haimen, People's Republic of China; ^3^ Department of Medical Oncology, Nanjing Medical University Affiliated Cancer Hospital of Jiangsu Province, Cancer Institution of Jiangsu Province, Nanjing, People's Republic of China; ^4^ Department of Oncology, The Affiliated Jiangyin Hospital, School of Medicine, Southeast University, Jiangyin, People's Republic of China

**Keywords:** pseudogene, DUXAP10, LSD1, proliferation, migration and invasion

## Abstract

Pseudogenes have been considered as non-functional transcriptional relics of human genomic for long time. However, recent studies revealed that they play a plethora of roles in diverse physiological and pathological processes, especially in cancer, and many pseudogenes are transcribed into long noncoding RNAs and emerging as a novel class of lncRNAs. However, the biological roles and underlying mechanism of pseudogenes in the pathogenesis of non small cell lung cancer are still incompletely elucidated. This study identifies a putative oncogenic pseudogene DUXAP10 in NSCLC, which is located in 14q11.2 and 2398 nt in length. Firstly, we found that DUXAP10 was significantly up-regulated in 93 human NSCLC tissues and cell lines, and increased DUXAP10 was associated with patients poorer prognosis and short survival time. Furthermore, the loss and gain of functional studies including growth curves, migration, invasion assays and in vivo studies verify the oncogenic roles of DUXAP10 in NSCLC. Finally, the mechanistic experiments indicate that DUXAP10 could interact with Histone demethylase Lysine specific demethylase1 (LSD1) and repress tumor suppressors Large tumor suppressor 2 (LATS2) and Ras-related associated with diabetes (RRAD) transcription in NSCLC cells. Taken together, these findings demonstrate DUXAP10 exerts the oncogenic roles through binding with LSD1 and epigenetic silencing LATS2 and RRAD expression. Our investigation reveals the novel roles of pseudogene in NSCLC, which may serve as new target for NSCLC diagnosis and therapy.

## INTRODUCTION

Lung cancer morbidity and mortality increased year by year. Non-small cell lung cancer (NSCLC) accounts for 80% of all lung cancers and can be divided into squamous cell carcinoma, adenocarcinoma, and large cell carcinoma, etc [1]. Despite significant advances in clinical, experimental oncology, and molecular targeting therapy for NSCLC, the prognosis for NSCLC remains poor, with overall 5-year survival rates as low as 15% [2, 3]. Lack of biomarkers for early diagnosis and effect target for therapy is still one of the most important challenge for NSCLC [4]. Therefore, a variety of studies on the mechanism of tumor progression, to improve the diagnosis, prevention and treatment of NSCLC patients, is essential.

Pseudogenes are considered as genomic loci that resemble parental genes, which are often considered to be nonfunctional ‘junk genes’ or ‘genomic fossil’ because they harbour mutations that abrogate their transcription or translation [5, 6]. Recently, however, the fast development and advance of next generation sequencing technique and the achievement of ENCODE project has revealed that numerous pseudogenes are indeed transcribed [7, 8]. In 2012, Shanker and his colleagues identified 2082 pseudogene transcripts based on next-generation sequencing data of 293 samples, among which 154 are highly tissue-specific and 218 expressed only in cancer samples [9, 10]. Moreover, Han et al detected 9925 pseudogene transcripts in 2808 samples across seven cancer types from The Cancer Genome Atlas (TCGA) RNA-seq data using a similar computational pipeline. This study also showed that many pseudogene transcripts are tissue and/or cancer-specific, and systematically revealed the potential of pseudogenes as prognostic and subtype biomarkers in cancers [11]. These findings indicating that aberrant pseudogenes may contribute to tumorigenesis, although their potential biological function and underlying mechanisms still remain elusive.

In recent years, a variety of cancers have been reported in which pseudogenes transcribe the diverse functions of long non-coding RNAs. For example, the pseudogene PTENP1 that highly homologous to the tumor suppressor gene PTEN was found to increase cellular levels of PTEN mRNA in prostate cancer through competitively binding to miR-17, miR-19, miR-21, miR-26 and miR-214 families. While reduced expression of PTENP1 released these miRNAs, which instead targeted PTEN mRNA causing reduced PTEN protein expression [12, 13]. In addition, OCT4 pseudogene OCT4pg1 is overexpressed in gastric cancer and its amplification is correlated with an aggressive phenotype and poor survival, while knockdown of OCT4pg1 promotes tumor growth and overexpression had anti-apoptotic effects [14]. Similarly, high expression of OCT4pg4 is correlated with poor prognosis in hepatocellular carcinoma, and increased OCT4pg4 expression released OCT4 from miRNA-145 mediated suppression of OCT4 translation, thereby increasing OCT4 protein levels and promoting growth and tumorigenicity [15, 16]. However, the role of pseudogene in NSCLC development is still completely not known, which need further investigation.

In this research, we identified a novel pseudogene DUXAP10 which is located in 14q11.2 and 2398 nt length. The biological function and expression pattern of DUXAP10 in cancers is not reported until now, and we found that DUXAP10 was significantly up-regulated in NSCLC tissues and cells. In addition, the inhibition and overexpression of functional assays were performed to explore the roles of DUXAP10 in NSCLC tumorigenesis, and mechanistic investigation was performed to reveal the molecular mechanism and underlying targets of DUXAP10 in NSCLC cells.

## RESULTS

### DUXAP10 is up-regulated and correlated with a poor prognosis in NSCLC

**Figure 1:**
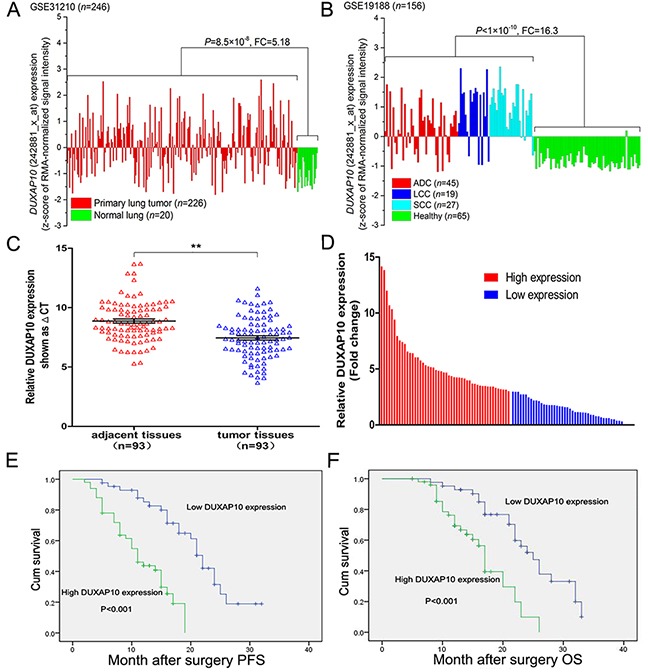
Relative DUXAP10 expression levels in NSCLC tissues and its clinical significance **A, B**. Relative expression of DUXAP10 in NSCLC tissues compared with normal tissue was analyzed by using GEO datasets GSE31210 and GSE19188. **C**. Relative expression of DUXAP10 in 93 pairs NSCLC tissues compared with corresponding non-tumor tissues was examined by qPCR, and normalized to GAPDH expression. **D**. The patients were divided into two groups according to DUXAP10 expression. **E**. Kaplan–Meier overall survival and progression-free survival curves according to DUXAP10 expression levels.

Firstly, we analyzed the profiles of NSCLC patient from Gene Expression Omnibus (GEO), and found that DUXAP10 was up-regulated in NSCLC tissues compared with normal lung tissues (Figure 1A and 1B). To determine whether DUXAP10 was overexpressed in NSCLC tissues, a total of 93 paired NSCLC tissue were evaluated for DUXAP10 expression using qPCR. The results showed that DUXAP10 was up-regulated in 81/93 (Figure 1C). To investigate the relationship between DUXAP10 levels and NSCLC patients clinicopathologic feature, we used the 3-fold changes value as a cutoff point to divide all patients into two groups: the high DUXAP10 group (n=50, fold-change ≥3.0), and the low DUXAP10 group (n=43, fold-change ≤3.0) (Figure 1D). Statistical analysis revealed that increased DUXAP10 expression were correlated with tumor size (p = 0.022), advanced pathological stage (P<0.001), and lymph node metastasis (p = 0.001). However, DUXAP10 expression was not associated with other factors including sex (p = 0.809) and age (p = 0.619) in NSCLC (Table 1).

**Table 1:** Correlation between DUXAP10 expression and clinicopathological characteristics of NSCLC patients (n = 93)

Characteristics	DUXAP10Low no. case(%)	DUXAP10High no. case(%)	PChi-squared test P-value
**Age(years)**			
>65	21(48.8)	27(54.0)	0.619
≤65	22(51.2)	23(46.0)	
**Gender**			
Male	26(60.4)	29(58.0)	0.809
Female	17(39.6)	21(42.0)	
**Histological subtype**			
Adenocarcinoma	21(48.8)	30(60.0)	0.281
Squamous cell carcinoma	22(51.2)	20(40.0)	
**TNM Stage**			
Ia + Ib	19(44.2)	4(8.0)	<0.001*
IIa + IIb	18(41.8)	25(50.0)	
IIIa	6(14.0)	21(42.0)	
**Tumor size**			
≤5cm	33(77.6)	27(54.0)	0.022*
>5cm	10(22.4)	23(46.0)	
**Lymph node metastasis**			
Negative	26(60.4)	13(26.0)	0.001*
Positive	17(39.6)	37(74.0)	
**Smoking History**			
Smokers	30(70.0)	32(64.0)	0.556
Never Smokers	13(30.0)	18(36.0)	

### Association between DUXAP10 expression and patient survival

Next, we investigated the association between DUXAP10 expression and prognosis in NSCLC patients by Kaplan-Meier survival analysis. Progression-free survival (PFS) was 46.5% for the low DUXAP10 group, and 32.0% for the high DUXAP10 group. Median survival time for the low DUXAP10 group was 22 months, and 11 months for the high DUXAP10 group (Figure 1E). As shown in Figure 1F, the overall survival rate over 2 years for the low DUXAP10 group was 35.6%, and 16.9% for the high DUXAP10 group. The median survival time for the low DUXAP10 group was 25 months, and 17 months for the high DUXAP10 group.

By univariate survival analysis, tumor size, lymph node metastasis, TNM stage and DUXAP10 expression level can be used as prognostic factors (Table 2). Moreover, multivariate Cox regression analyses showed that expression of DUXAP10 (p=0.013), along with TNM stage (P=0.023) and lymph node metastasis(p=0.029), were independent prognostic factors for NSCLC patients (Table 2).

**Table 2:** Univariate and multivariate analysis of over-survival in NSCLC patients (n=93)

Variables	Univariate analysis			Multivariate analysis		
	HR	95% CI	p value	HR	95% CI	p value
age	1.148	0.644-2.045	0.639			
gender	1.317	0.738-2.349	0.351			
smoker	1.281	0.716-2.291	0.404			
Histological subtype	1.410	0.789-2.517	0.246			
Chemotherapy	0.618	0.341-1.119	0.112			
Tumor size	2.054	1.148-3.676	0.015*	1.812	0.979-3.353	0.058
Lymph node metastasis(No vs. Yes)	2.463	1.327-4.571	0.004*	2.126	1.079-4.190	0.029*
TNM stage (IIIa vs. I or II)	2.589	1.410-4.752	0.002*	2.097	1.106-3.974	0.023*
DUXAP10 expression(High vs. Low)	3.481	1.808-6.701	<0.001*	2.567	0.979-3.353	0.013*

HR, hazard ratio; 95 % CI, 95 % confidence interval, * Overall P < 0.05.

### Modulation of DUXAP10 expression in NSCLC cells

**Figure 2:**
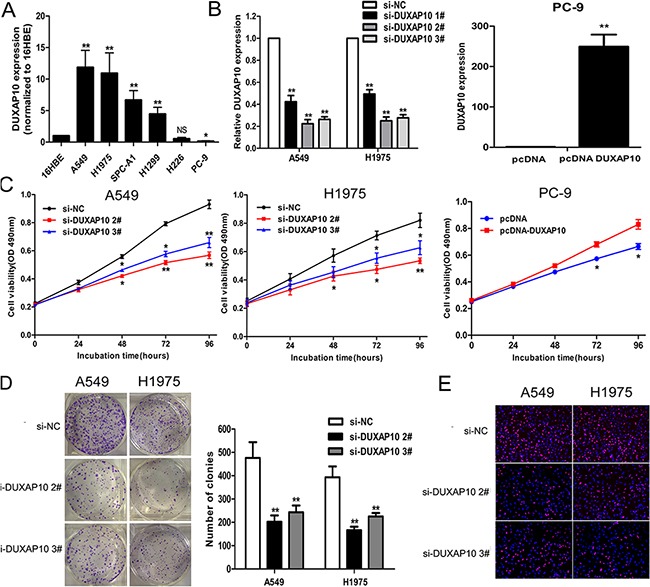
Effects of DUXAP10 on NSCLC cell proliferation *in vitro* **A**. DUXAP10 expression levels of NSCLC cell lines (A549, H1975, SPC-A1, H1299, H226 and PC-9), compared with that in normal human bronchial epithelial cells (16HBE). **B**. A549 and H1975 cells were transfected with si-DUXAP10, PC-9 cells were transfected with pCDNA-DUXAP10. **C**. MTT assays were performed to determine the cell viability for si-DUXAP10-transfected A549 and H1975, and PC-9 cells transfected with pCDNA-DUXAP10. **D, E**. Colony-forming assays and EDU staining assays were used to determine the proliferation of si-DUXAP10-transfected A549 and H1975 cells. *P<0.05, **P<0.01.

In order to explore the biological function of DUXAP10 in NSCLC cells, we firstly evaluate the expression of DUXAP10 in various NSCLC cell lines using qPCR assays. As shown in Figure 2A, four cell lines (A549, H1975, SPC-A1 and H1299) expressed higher levels of DUXAP10 compared with the normal bronchial epithelial cell line (16HBE). In contrast, the relative expression level of DUXAP10 was lower in PC-9 cell lines. Next, DUXAP10 expression was knockdown in A549 and H1975 cells by transfection with siRNAs, and over-expressed by transfected with pCDNA-DUXAP10 vector. Furthermore, the results showed that DUXAP10 expression was reduced by approximately 77.6% or 73.6% in A549 cells (Figure 2B), and up-regulated approximately 249.5-folds compared with control in PC-9 cells.

### Effect of DUXAP10 on NSCLC cell proliferation and cell cycle progression

To identify the role of DUXAP10 in NSCLC cells, we performed a series of functional loss and gain assays. MTT and colony formation assays showed reduced proliferation of A549 and H1975 cells transfected with si-DUXAP10 compared to control cells (Figure 2C and 2D), while DUXAP10 ovexpression promoted PC-9 cells proliferation (Supplementary Figure S1). In addition, EdU staining assays showed the same results. (Figure 2E)

**Figure 3:**
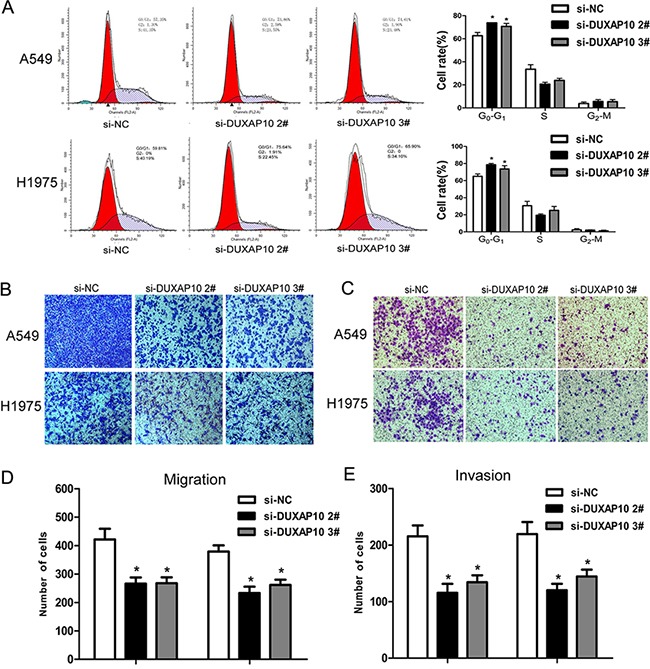
Knockdown of DUXAP10 inhibited cell cycle and cell migration and invasion *in vitro* **A**. Flow cytometry assays were performed to analysis the cell cycle progression when NSCLC cells transfected with si-DUXAP10. The bar chart represented the percentage of cells in G0/G1, S, or G2/M phase, as indicated. All experiments were performed in biological triplicates with three technical replicates. **B** to **E**. Effect of knockdown of DUXAP10 on cell migration and invasion. Data are presented as mean ± SD. *P<0.05, **P<0.01.

To further examine whether the effect of DUXAP10 on NSCLC cell proliferation exhibits cell cycle arrest, we used flow cytometry to analyze cell cycle progression. The results showed that A549 or H1975 cells transfected with si-DUXAP10 had significant cell cycle arrest in G1 / G0 phase and a decrease in G2 / S phase (Figure 3A). However, the flow cytometry analysis showed that knockdown of DUXAP10 had no effect on NSCLC cells apoptosis. These data indicate that DUXAP10 could promote the cell cycle progression and proliferation phenotype of NSCLC cells.

### Knockdown of DUXAP10 inhibits NSCLC cells migration and invasion

Migration and metastasis of cancer cells is an important factor in the progress of cancer, we conducted a transwell test of DUXAP10 on NSCLC cell migration and invasion. The results revealed that decreased DUXAP10 impeded the NSCLC cells migration and invasion compared with controls (Figure 3B to 3E). These results suggested that knockdown of DUXAP10 had tumor-suppressive function that could inhibit NSCLC cells migration and invasion.

### Knockdown of DUXAP10 inhibits NSCLC cell tumorigenesis *in vivo*

**Figure 4:**
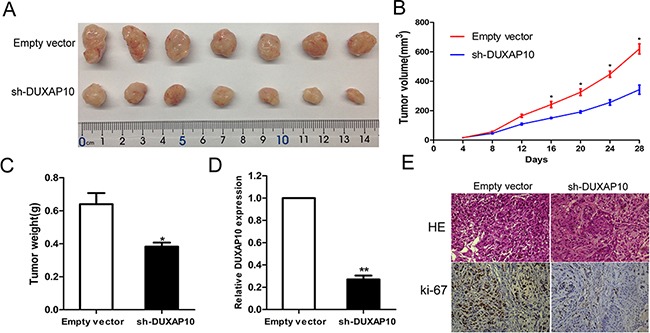
The stable DUXAP10 knockdown A549 cells were used for the *in vivo* study **A** and **B**. The nude mice carrying tumors from respective groups were shown and tumor growth curves were measured after the injection of A549 cells. Tumor volume was calculated every 4 days. **C**. Tumor weights are represented as means of tumor weights ±S.D. **D**. qPCR assay was performed to determine the average expression of DUXAP10 in xenograft tumors. **E**. Tumors developed from sh-DUXAP10 transfected A549 cells showed lower Ki67 protein levels than tumors developed by control cells. Upper: H & E staining; Lower: immunostaining.

To further study the effect of DUXAP10 expression on tumor growth in vivo, sh-DUXAP10 or empty vector transfected A549 cells were inoculated subcutaneously in male nude mice. Twenty-eight days after injection, the tumor size of the sh-DUXAP10 group was significantly smaller compared with the control group (Figure 4A and 4B). The tumor weight of sh-DUXAP10 group was also significantly lower than that in the control group (Figure 4C). Next, qPCR assays determined that DUXAP10 expression levels were down-regulated in tumor tissues collected from sh-DUXAP10 group compared with control group (Figure 4D). In addition, immunohistochemistry (IHC) analysis confirmed that the tumors formed from A549/sh-DUXAP10 cells displayed less positive Ki-67 staining than those of control cells (Figure 4E). These results indicated that inhibition of DUXAP10 could suppress tumor progression *in vivo*.

### LATS2 and RRAD are key downstream mediator of DUXAP10 in NSCLC cells

**Figure 5:**
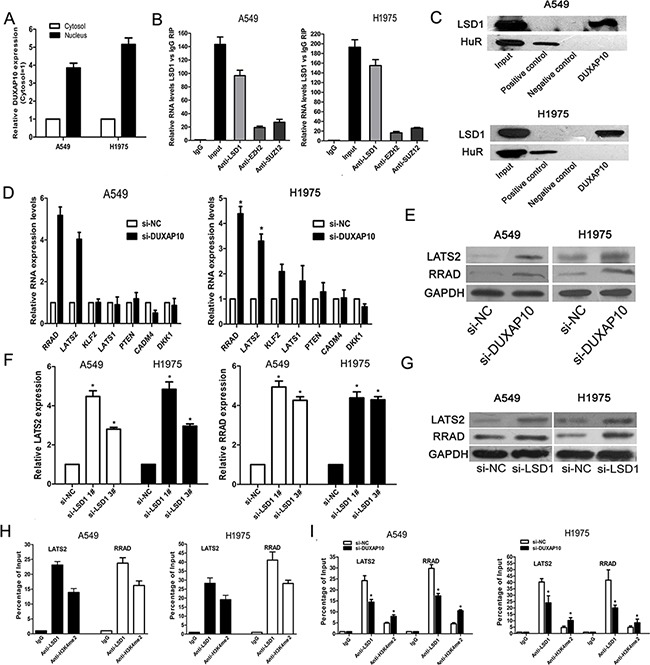
DUXAP10 could inhibit LATS2 and RRAD expression **A**. DUXAP10 expression levels in cell cytoplasm or nucleus of NSCLC cell lines A549 and H1975 were detected by qPCR. GAPDH was used as a cytosol marker and U1 was used as a nucleus marker. **B**. RIP with rabbit monoclonal anti-LSD1, rabbit monoclonal anti-EZH2, rabbit monoclonal anti-SUZ12, preimmune IgG, or 10% input from A549 and H1975 cell extracts. RNA levels in immunoprecipitates were detected by qPCR. Expression levels of DUXAP10 RNA are presented as fold enrichment in LSD1 relative to IgG immunoprecipitates. **C**. RNA pulldown and western blotting assays were performed and the results revealed that DUXAP10 could bind to LSD1. **D** and **E**. The qPCR and western blot assay were conducted to detect the levels of LATS2 and RRAD mRNA in A549 and H1975 cells transfected with si-DUXAP10 and results are expressed relative to the corresponding values for control cells. **F** and **G**. QPCR and Western blot assays were used to detect the LATS2 and RRAD expression both in mRNA and protein levels in A549 and H1975 cells transfected with si-LSD1. **H** and **I**. ChIP–qPCR of LSD1 occupancy and H3K4-2me binding in the LATS2 and RRAD promoter in A549 and H1975 cells, and IgG as a negative control. At 48h after transfection, ChIP–qPCR of LSD1 occupancy and H3K4-2me binding in the LATS2 and RRAD promoter in A549 and H1975 cells treated with si-DUXAP10 or scrambled siRNA. *P<0.05, **P<0.01.

We detected the distribution of DUXAP10 in NSCLC cells by subcellular fractionation assays. The results showed that DUXAP10 mostly located in nucleus (Figure 5A and Supplementary Figure S2A). Then, we chose several RNA binding proteins which can regulate targets expression at transcriptional levels, and performed RIP assays to investigate their potential interaction with DUXAP10 in NSCLC cells. The results showed that DUXAP10 enrichment in LSD1-RNA precipitates (Figure 5B), but the enrichment was not observed in other protein-RNA precipitates. Furthermore, we conducted RNA-pulldown assays in A549 and H1975 cells to determine whether LSD1 is associated with DUXAP10. The results revealed that LSD1 could directly bind with DUXAP10 (Figure 5C).

To further explore the underlying target genes of DUXAP10 in NSCLC cells, we analyzed previously published gene expression profile downstream of LSD1 in breast cancer cells and other known LSD1 targets. The qPCR results showed that DUXAP10 knockdown did not affect the expression of KLF2 et al. genes in A549 and H1975 cells, but increased the expression of RRAD and LATS2 (Figure 5D). To further verify this result, we conducted western blot analysis and revealed that Ras-related associated with diabetes (RRAD) and Large tumor suppressor 2 (LATS2) protein levels was also increased in si-DUXAP10 transfected cells (Figure 5E).

### DUXAP10 represses RRAD and LATS2 transcription by interacting with LSD1

To determine whether DUXAP10 repressed RRAD and LATS2 expression via interacting with LSD1 in NSCLC cells, we evaluated their expression after knockdown of LSD1 in NSCLC cells. Interestingly, knockdown of LSD1 also upregulated RRAD and LATS2 expression(Figure 5F and 5G). To further determine whether LSD1 could directly bind the promoter region of RRAD and LATS2, we designed four pairs of primers across 2000 bp of the promoter region. CHIP assays confirmed that LSD1 could bind to the RRAD and LATS2 promoter region (Figure 5H). Moreover, knockdown of DUXAP10 reduced LSD1 binding to RRAD and LATS2 promoter regions (Figure 5I).

### Overexpress of LATS2 and RRAD is partly involved in the oncogenic function of DUXAP10

**Figure 6:**
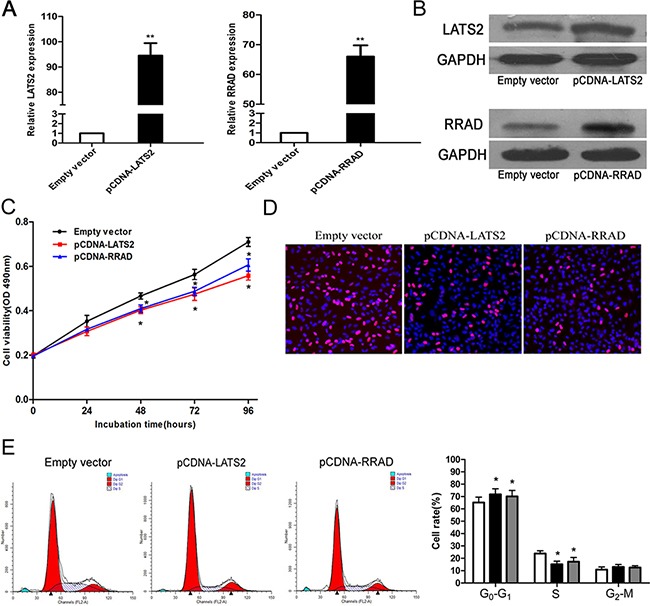
Effect of LATS2 and RRAD of overexpression on A549 cell *in vitro* **A, B**. The mRNA levels and protein levels of LATS2 and RRAD in A549 cells transfected with pCDNA–LATS2 or pCDNA-RRAD was detected by qPCR analysis. **C, D**. MTT assays and Edu staining assays were used to determine the cell viability. Values represent the mean ± s.d. from three independent experiments. **E**. Cell cycle was analyzed by flow cytometry. The bar chart represents the percentage of cells in G1–G0, S, or G2–M phase, as indicated. *P < 0.05 and **P < 0.01.

We performed an overexpression functional assay to further investigate whether LATS2 and RRAD are involved in the promotion of DUXAP10-induced proliferation of NSCLC cells. LATS2 and RRAD expression showed rising trends in A549 cells transfected with pCDNA-LATS2 and pCDNA-RRAD compared with control cells which are conducted by q-PCR and western blot assays (Figure 6A and 6B). MTT and EdU assays demonstrated that the NSCLC cell viability was inhibited upon overexpression of RRAD and LATS2 (Figure 6C and 6D). Furthermore, our results showed that ectopic expression of LATS2 or RRAD could also induce G1–G0 phase arrest (Figure 6E).

**Figure 7:**
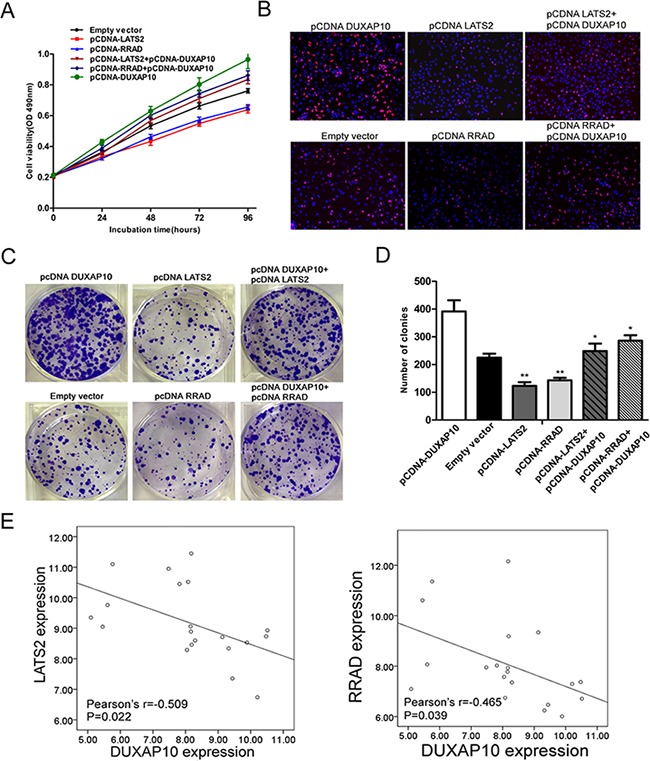
DUXAP10 negatively regulates expression of LATS2 and RRAD by rescue assays **A, B, C, D**. MTT, edu and colony formation assays were used to determine the cell proliferation ability for A549 cells transfected with pCDNA-DUXAP10 and pCDNA-LATS2 and pCDNA-RRAD and co-transfected with pCDNA-DUXAP10 and pCDNA-LATS2 or pCDNA-DUXAP10 and pCDNA-RRAD. **E**. QPCR analyzed the LATS2 and RRAD mRNA levels in 20 pairs NSCLC tissues and found that there was a significantly negative correlation between DUXAP10 and RRAD or LATS2. Values represent the mean ± s.d. from three independent experiments.

Moreover, we conducted rescue assays to determine whether LATS2 and RRAD involved in DUXAP10 contributions to NSCLC cell proliferation. A549 cells were co-transfected with pCDNA-DUXAP10 and pCNDA-LATS2 or pCDNA-RRAD, and pCDNA-LATS2 or pCDNA-RRAD transfection could partly rescue pCDNA-DUXAP10 decreased LATS2 or RRAD expression. The MTT, EdU staining assays and colony formation showed that co-transfection could partly reverse pCDNA-DUXAP10 induced growth (Figure 7A to 7D). Finally, we analyzed the correlation between DUXAP10 and LATS2 and RRAD expression in 20 pair NSCLC tissues, and found that there was a significantly negative correlation between DUXAP10 and RRAD or LATS2 (Figure 7E). These data suggest that DUXAP10 may exert an oncogenic effect in NSCLC cells, in part, by inhibiting RRAD and LATS2 expression.

## DISCUSSION

Over the past decade, remarkable progress has been made in establishing lncRNAs as critical regulators of various biological processes, and aberrant lncRNAs expression are implicated in the development of multiple cancers [9, 17–19]. Recently, the Competitive Endogenous RNA (ceRNA) paradigm has refocused the attention on a new sub-class of lncRNA genes-pseudogenes, which shares high sequence homology with their parental genes enables them to participate in posttranscriptional regulation through competition for shared miRNAs [20, 21]. Importantly, pseudogenes also involved in cancers development by function as ceRNAs. The pseudogene BRAFP1 regulates BRAF expression through functions as a ceRNA and competitive binding of miR-30a, miR-182, miR-876, and miR-590, which finally Induces lymphoma *in vivo* [22]. In addition, pseudogene can also recruitment of regulatory proteins to complementary sites to modulate chromatin remodeling and transcription or competition for RNA-binding proteins or the translation machinery [23]. However, whether pseudogene could regulate other genes not their parental genes in cancers is not clear.

In the present study, we found that pseudogene DUXAP10 is significantly overexpressed in NSCLC tissues and cells. Knockdown of DUXAP10 inhibited NSCLC cell proliferation, migration and invasion, while DUXAP10 overexpression promoted NSCLC cells proliferation, migration and invasion. The *in vivo* investigation also showed that knockdown of DUXAP10 impaired tumor growth. Furthermore, mechanism investigation showed that there are no sequence of DUXAP10 homology with its parental gene. However, further studies indicated that DUXAP10 exerted oncogenic functions in human NSCLC cells by interacting with LSD1 and repressing RRAD and LATS2 expression at transcriptional level. Our study provided evidence that pseudogene could also regulate other genes not their parental genes through interacting with histone protein modification enzymes.

RRAD is a member of the Ras-like small GTPase family, which is initially identified as a gene associated with Type II diabetes [24]. Recent studies have revealed that RRAD also has tumor suppressive function in many types of cancers [25], and RRAD is frequently down-regulated in multiple cancers including lung cancer, due to the hypermethylation of its promoter region [26]. Similarly, hypermethylation of promoter also contributes to another tumor suppressor LATS2 downregulation in cancer cells [27, 28]. LATS2, a member of the LATS tumor suppressor family, has been identified as a new regulator of cellular homeostasis [29, 30]. Importantly, LATS2 is also downregulated in NSCLC and its decreased expression promoted NSCLC cells growth and motility [31, 32]. Meanwhile, ectopic RRAD expression could inhibited the nasopharyngeal carcinoma cell growth and cell migration, and negatively regulates the NF-κB signaling to inhibit the GLUT1 translocation and the Warburg effect in lung cancer cells [33, 34]. In this study, we also found that RRAD and LATS2 expression is downregulated in NSCLC, and their overexpression impeded cell proliferation. Importantly, pseudogene DUXAP10 interacting with LSD1 mediated H3K4me2 demethylation might partly invovled in the downregulation of RRAD and LATS2 in NSCLC cells.

Previous studies have demonstrated that pseudogene also could regulated target genes expression through competing sponging specific miRNAs. However, whether DUXAP10 could regulate other possible targets and the mechanism that underlie regulatory behaviors were not investigated in this study, which needs to be further investigated. In conclusion, our study showed for the first time that pseudogene DUXAP10 expression is up-regulated in NSCLC tissues and cells, indicating that its overexpression may be a negative prognostic factor for NSCLC patients. Knockdown of DUXAP10 exerted tumor-suppressive functions through reducing cell proliferation, migration as well as inducing apoptosis in NSCLC, while DUXAP10 overexpression promoted cell proliferation, and migration. Furthermore, DUXAP10 mediated the oncogenic effects is partially through its epigenetic silencing of the RRAD and LATS2 expression by binding with LSD1. Our findings further the understanding of NSCLC pathogenesis, and facilitate the development of pseudogene-directed diagnostics and therapeutics against this disease.

## MATERIALS AND METHODS

### Psudogene expression from GEO DataSets

Psudogene expression from GEO DataSets NSCLC gene expression data were obtained fromGEODataSets (GDS). Two independent data sets from GSE31210, GSE19188 were included in this study. The raw CEL files were downloaded from GEO database and normalized using Robust Multichip Average (RMA). After we downloaded probe sequences from GEO or microarray manufacturers, blastt2.2.30 was used to reannotates probe on GENCODE Release 21 sequence databases for psudogene and mRNA. For multiple probes corresponding to one gene, maximum normalized signal was selected to generate expressions of psudogene and mRNA. Rank-sum test according to experimental design was employed as differential expression calling method, followed by the Benjamini–Hochberg (FDR) adjustment.

### Cell lines

Six NSCLC cell lines (A549, H1975, SPC-A1, PC-9, H1299, H226) and the normal bronchial epithelial cell line 16HBE were obtained from the Institute of Biochemistry and Cell Biology of the Chinese Academy of Sciences (Shanghai, China). A549, H1975 and H1299 cells were cultured in RPMI-1640, and 16HBE, PC9 and SPC-A1 cells were cultured in DMEM medium (GIBCO-BRL) supplemented with 10% fetal bovine serum (Gibco),100 U/ml penicillin sodium, and 100 mg/ml streptomycin sulfate at 37°C in a humidified air atmosphere containing 5% CO2.

### Tissue specimens and clinical data collection

We obtained 93 paired NSCLC and adjacent non-tumor lung tissues from patients who underwent surgery at The First and Second Affiliated Hospital of Nanjing Medical University from 2011 to 2012. These patients were diagnosed with NSCLC (stages I, II, and III) based on histopathological evaluation. The clinicopathological characteristics of the NSCLC patients are summarized in Table 1. The patients did not receive any local or systemic treatment before operation. The ethics committee of The Second Affiliated Hospital of Nanjing Medical University approved the study protocol.

### RNA extraction and qPCR assays

Total RNA were etracted with TRIZOL reagent from tissue samples or cells according to the manufacturer's instructions. Total RNA (500 ng) was reverse transcribed in a final volume of 10 μl using random primers under standard conditions with the PrimeScript RT Reagent Kit (TaKaRa, Dalian, China). To analysis DUXAP10 expression levels, we used SYBR Premix Ex Taq (TaKaRa) following the manufacturer's instructions. Primer sequences are listing in Supplementary Table S1. Real-time PCR was performed in triplicate on an ABI 7500, and data were calculated using the comparative cycle threshold (CT) (2^−ΔΔCT^) method.

### RNA interference

A549 and H1975 cells were transfected with siRNA using Lipofectamine 2000 (Invitrogen, USA) and the cells were incubated for 48 h before use in assays. The siRNA sequences are listed in Supplementary Table S1.

### Plasmid generation

The LATS2 and RRAD sequence were respectively synthesized and subcloned into the pCDNA3.1 vector (GENECHEM, Shanghai, China) to generate the pCDNA-LATS2 vector and pCDNA-RRAD vector for ectopic expression in cells. pCDNA3.1 vector was used as a control. We adopted qPCR assay to evaluate expressions of LATS2 and RRAD.

### Cell viability assays

Cell viability was tested by using a Cell Proliferation Reagent Kit I ( MTT; Roche Applied Science). The A549 and H1975 cells transfected with si-DUXAP10, and PC-9 cells transfected with pCDNA-DUXAP10 were grown in 96-well plates. Cell viability was tested every 24 h following the manufacturer's protocol. For colony formation assay, transfected cells (n=1000) were placed in six-well plates and maintained in proper medium containing 10% FBS for 2 weeks. Visible colonies were then counted. For each treatment group wells were assessed in triplicate.

### Cell migration and invasion assays

Transwells (Corning, Tewksbury, MA, USA, 8.0-μm pores) were used to measure migration and invasion. For migration assays, 4×104 cells were suspended in 300 μl of DMEM or 1640 containing 1% fetal bovine serum and transferred to the upper chamber. For the invasion assays, 1 × 105 cells in 1% fetal bovine serum medium were placed into the upper chamber of an insert coated with Matrigel (Sigma-Aldrich). Medium containing 10% FBS was added to the lower chamber. After incubation for 24 h, the cells that had migrated or invaded through the membrane were stained with methanol and 0.1% crystal violet, imaged, and counted using an IX71 inverted microscope (Olympus, Tokyo, Japan).

### Flow cytometric analysis

We harvested A549 and H1975 cells that were transfected with si-DUXAP10 for 48 h. Cells were stained with propidium oxide using the CycleTEST PLUS DNA Reagent Kit (BD Biosciences) following the manufacturer's protocol and analyzed by FACScan. The percentages of cells in G0–G1, S, and G2-M phases were counted and compared.

### Western blot assay and antibodies

Protein lysates were separated by 12% SDS-polyacrylamide gel electrophoresis (SDS-PAGE) and transferred to 0.22-μm NC membranes (Sigma) and incubated with specific primary antibodies. Anti-LATS2 (1:1000) was purchased from SAB, RRAD antibodies (1:1000) were purchased from Abcam, GAPDH antibody was used as a control.

### EdU assay

Proliferating cells were assessed using a 5-ethynyl-2-deoxyuridine (EdU) labeling/detection kit (Ribobio, Guangzhou, China), in accordance with the manufacturer's protocol. Briefly, A549 or H1975 cells were cultured in 96-well plates at 5 × 103 cells per well and transfected with plasmid DNA or siRNA for 48 h. Then, 50 μM EdU labeling medium was added to the cell culture and incubated for 2 h at 37°C under 5% CO2. Next, the cultured cells were fixed with 4% paraformaldehyde (pH 7.4) for 30 min and treated with 0.5% Triton X-100 for 20 min at room temperature. After washing with phosphate-buffered saline (PBS), the samples were stained with anti- EdU working solution at room temperature for 30 min. Subsequently, the cells were incubated with 100 μL of Hoechst 33342 (5 μg/mL) at room temperature for 30 min, followed by observation under a fluorescent microscope. The percentage of EdU-positive cells was calculated from five random fields in three wells.

### Tumor formation assay in a nude mouse model

Male athymic BALB/c mice (5 weeks old) were maintained under specific pathogen-free conditions and manipulated according to protocols approved by the Shanghai Medical Experimental Animal Care Commission. A549 cells stably transfected with sh-DUXAP10 or empty vector were harvested at a concentration of 2.5 × 107 cells/ml, and 0.1 ml was subcutaneously injected into the flanks of the nude mice, at one injection per mouse. Tumor growth was monitored, and tumor sizes and weights were measured every four days. Tumor volume was calculated using the formula, volume = (length × width2 × 0.5). At 28 days after injection, the mice were killed and tumor weights were measured. The primary tumors were excised and tumor tissues were used for qRT-PCR analysis of DUXAP10 expression levels and immunostaining analysis of Ki-67 protein expression.

### Subcellular fractionation

The separation of nuclear and cytosolic fractions was performed using the PARIS Kit (Life Technologies, Carlsbad, CA, USA) according to the manufacturer's instructions.

### RNA immunoprecipitation (RIP) assays

A549 and H1975 cells were lysed for immuno precipitation (IP) of endogenous LSD1 from whole-cell extracts. The protein A Sepharose beads incubated negative antibody IgG and interested antibody LSD1 (Milipore, USA) for 30 minutes at 4°C. Then, the supernatants of whole-cell extracts were incubated with treated-beads for 6h at 4°C. We used the wash buffer to wash the beads for 6 times. To isolate the RNA-protein complexes from beads, the beads incubated with 0.1% SDS/0.5mg/ml Proteinase K for 30 minutes at 55°C. The qPCR assays were used to further evaluate the LSD1 isolated from the IP materials.

### Chromatin immunoprecipitation (ChIP) assays

ChIP assays were conducted using the EZ-CHIP KIT according to the manufacturer's instructions (Millipore, USA). LSD1 antibody was obtained from Abcam. H3 trimethyl Lys 4 antibody was from Millipore. Quantification of immunoprecipitated DNA was performed using qPCR with SYBR Green Mix (TaKaRa). ChIP data was calculated as a percentage relative to the input DNA.

### RNA pulldown assays

The pCDNA3.1-DUXAP10 vector was cutted by restriction enzymes Nru I and treated with RNase-free Dnase I (Biolabs, New England). DUXAP10 was transcribed from cutted-vector by mMESSAGE mMACHINE T7® Kit (Ambion, USA) and purified with an RNeasy Mini Kit (Qiagen, Valencia, CA) *in vitro*. We biotinlabeled the 3′ end of lncRNA-DUXAP10 referencing to the instruction of Pierce RNA 3´ End Desthiobiotinylation Kit (Thermo Scientific, USA). One milligram of protein from A549 and H1975 cell extracts were then mixed with 50 pmol of biotinylated RNA, incubated with 50μL of magnetic beads for 1h at 4°C (Thermo Scientific, USA). The RNA-protein complex was isolated from magnetic beads by Biotin Elution Buffer and boiled in sodium dodecyl sulfate (SDS) buffer for 5 minuts. The retrieved protein was detected using the standard western blot technique.

### Immunohistochemistry (IHC)

The primary tumors were immunostained for Ki-67 as previously described.

### Statistical analysis

All statistical analyses were performed using SPSS 20.0 software (IBM). The significance of differences between groups was estimated by the Student's t-test, Wilcoxon test, or chi-squared test. Progression-free survival (PFS) and overall survival (OS) rates were calculated by the Kaplan–Meier method with the log-rank test applied for comparison. The date of survival was evaluated by univariate and multivariate Cox proportional hazards models. Variables with P < 0.05 in univariate analysis were used in subsequent multivariate analysis on the basis of Cox regression analyses. Kendall Tau-b and Pearson correlation analyses were conducted to investigate the correlation between DUXAP10 and LATS2 and RRAD expressions. Two-sided P values were calculated, and a probability level of 0.05 was chosen for statistical significance.

## SUPPLEMENTARY FIGURES AND TABLE




