# Supplementary material for: The pseudogene DUXAP10 promotes an aggressive phenotype through binding with LSD1 and repressing LATS2 and RRAD in non small cell lung cancer
Source: Oncotarget. 2016 Dec 24;8(3):5233–46. doi: 10.18632/oncotarget.14125 (PMC5354904; doi:10.18632/oncotarget.14125)
Supplement: Supplementary file 1 [file oncotarget-08-5233-s001.pdf]

The pseudogene DUXAP10 promotes an aggressive phenotype through binding with LSD1 and repressing LATS2 and RRAD in non small cell lung cancer

SUPPLEMENTARY FIGURES AND TABLE

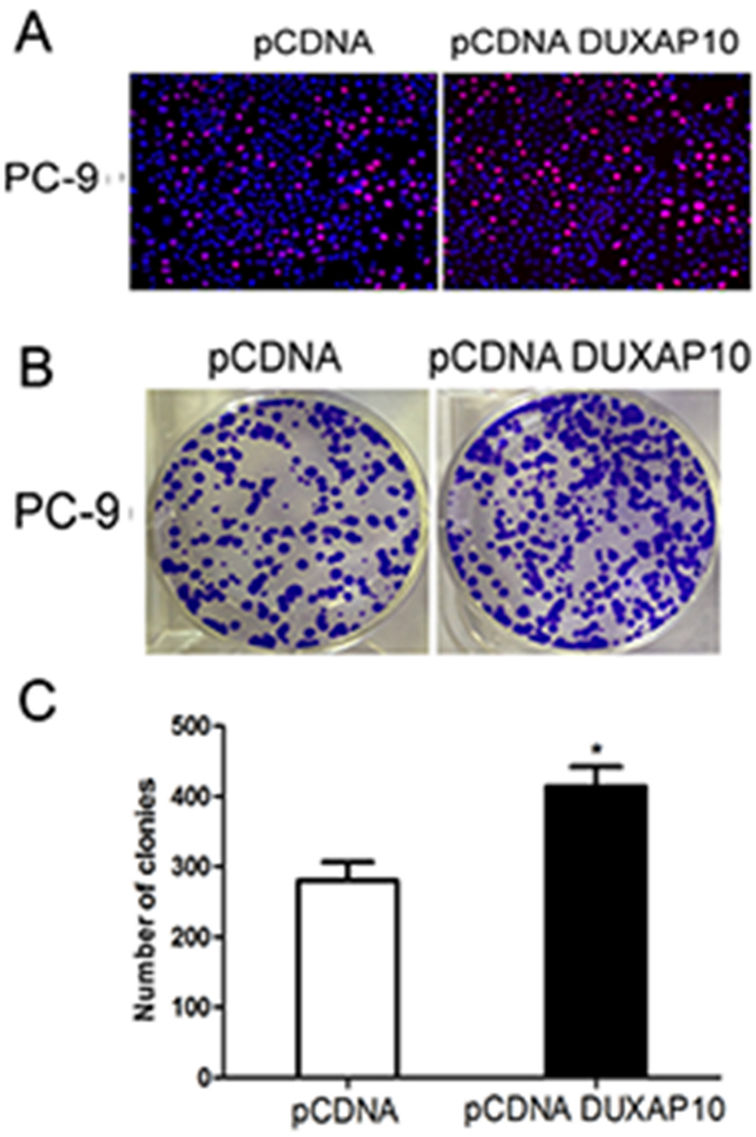

Supplementary Figure S1: Overexpression of DUXAP10 on NSCLC cell proliferation in vitro. A-C. EDU staining assays and colony-forming assays were used to determine the proliferation of pcDNA-DUXAP10-transfected PC9 cells.

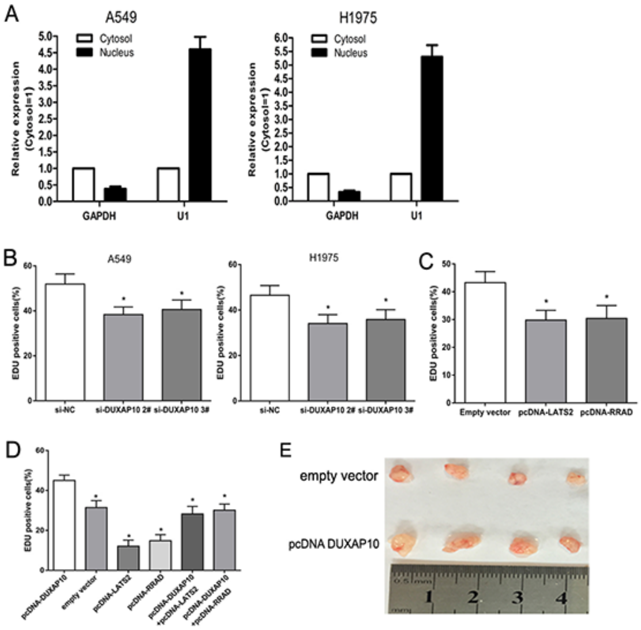

Supplementary Figure S2: Quantification of Figure 2E, 6D, 7B, and in vivo study of DUXAP10 overexpression. **A.** GAPDH and U1 expression levels in cell cytoplasm or nucleus of NSCLC cell lines A549 and H1975 were detected by qPCR. GAPDH was used as a cytosol marker and U1 was used as a nucleus marker. **B-D.** Quantification of Figure 2E, 6D, 7B. **E.** The nude mice carrying tumors from respective groups were shown after the injection of PC9 cells.

Supplementary Table S1: Sequence of primers and siRNA.

See Supplementary File 1
