# Supplementary material for: The pseudogene DUXAP10 promotes an aggressive phenotype through binding with LSD1 and repressing LATS2 and RRAD in non small cell lung cancer
Source: Oncotarget. 2016 Dec 24;8(3):5233–46. doi: 10.18632/oncotarget.14125 (PMC5354904; doi:10.18632/oncotarget.14125)
Supplement: Supplementary file 2 [file oncotarget-08-5233-s002.doc]

| Si-RNA |  |
| --- | --- |
| si-DUXAP10 1# | GGAACUUCCCAAACCUCCAUGAUUU |
| si-DUXAP10 2# | CAGCAUACUUCAAAUUCACAGCAAA |
| si-DUXAP10 3# | AGUUGUUUGUUAGAAUACUGGUGCU |
| si-LSD1 1# | CAUUUGAGGCUACUCUCCAACAAUU |
| si-LSD1 2# | CAAAGGAUGGGAUUUGGCAACCGGA |
| si-LSD1 3# | GGUCUUAUCAACUUCGGCAUCUAUA |
|  |  |
| Primers |  |
| GAPDH F | GGGAGCCAAAAGGGTCAT |
| GAPDH R | GAGTCCTTCCACGATACCAA |
| DUXAP10 F | CTGTAGGAGGCCAAGACAGG |
| DUXAP10 R | CATTGTCTCAAGGTCTGCTGAA |
| LATS2 F | ACCCCAAAGTTGGACCTTA |
| LATS2 R | CATTTGCCGGTTCACTTCTGC |
| RRAD F | TTTACAAGGTGCTGCTGCTGGG |
| RRAD R | TGCCGCTGATGTCTCAATGAAC |
| LSD1 F | CAAGTGTCAATTTGTTCGGG |
| LSD1 R | TTCTTTGGGCTGAGGTACTG |
| LATS1 F | TTACCAAGATCCTCGACGAGAG |
| LATS1 R | CACATTCCCTGGTTTCATGCT |
| KLF2 F | TTCGGTCTCTTCGACGACG |
| KLF2 R | TGCGAACTCTTGGTGTAGGTC |
| PTEN F | TGGATTCGACTTAGACTTGACCT |
| PTEN R | GGTGGGTTATGGTCTTCAAAAGG |
| CADM4 F | GCCGTCTGCACCAGTATGAT |
| CADM4 R | CTGGAAACGCTCATCCTTCAA |
| DKK1 F | CCTTGAACTCGGTTCTCAATTCC |
| DKK1 R | CAATGGTCTGGTACTTATTCCCG |
|  |  |
| ChIP-qRT-PCR primers |  |
| LATS2 P366 F | GGCAGGAGGATGGCTTGA |
| LATS2 P366 R | GCCCTACTGGCATTACC |
| LATS2 P802 F | GACTGAAGGCTGGGTGAG |
| LATS2 P802 R | ATGGGAAACAGTAGTAAAGG |
| LATS2 P1583 F | CCACTGTCCCAATAGCCG |
| LATS2 P1583 R | CCTGAAGGAGCCACCAAG |
| LATS2 P1060 F | CCCTGCTTCTGTCCCTGTA |
| LATS2 P1060 R | CCAGCAAGTGAGGTCGTG |
|  |  |
| RRAD P475 F | GGAAGTGTGTGGTGGCTC |
| RRAD P475 R | GCTCGGTAAGATTAGGGC |
| RRAD P722 F | GCCAAGGAAACTGATGCA |
| RRAD P722 R | ACAAGTGTGGTGGGGGTG |
| RRAD P1152 F | GGCAACTTCCAGTCCAGG |
| RRAD P1152 R | TCAGTCCCCATCCCACCT |
| RRAD P1745 F | ACCTCGCTCTCTCTCTCCTTCTC |
| RRAD P1745 R | ACCCTCTTCCTCGGACCTTACAT |
